# Salinity stress reduces tree reproductive allocation in coastal forests

**DOI:** 10.17912/micropub.biology.001778

**Published:** 2026-02-13

**Authors:** Juan Ignacio Martínez, Aliya Khan, Keryn Gedan

**Affiliations:** 1 Biological sciences, The George Washington University; 2 Earth and Planetary Sciences, University of California, Santa Cruz, Santa Cruz, California, United States

## Abstract

Plants adjust reproductive allocation in response to environmental changes to maximize fitness. As sea level rise increases salinity in coastal forests, we examined its effects on seed rain in two tree species. *Pinus taeda* showed a median 52% reduction relative investment in pine cone production in high-salinity areas relative to low salinity areas. *Juniperus virginiana* decreased relative investment in seed production by 64%, though with higher uncertainty. These results reveal that salinity stress reduces tree allocation to reproduction in coastal forests, and suggests species-specific reproductive responses to environmental change, with implications for coastal forest dynamics under rising salinity.

**Figure 1. Litter production, cone/berry production, and relative reproductive investment across elevation zones for two tree species. High , Mid and Low represent forest zones along an elevation gradient that correlates with salinity:**
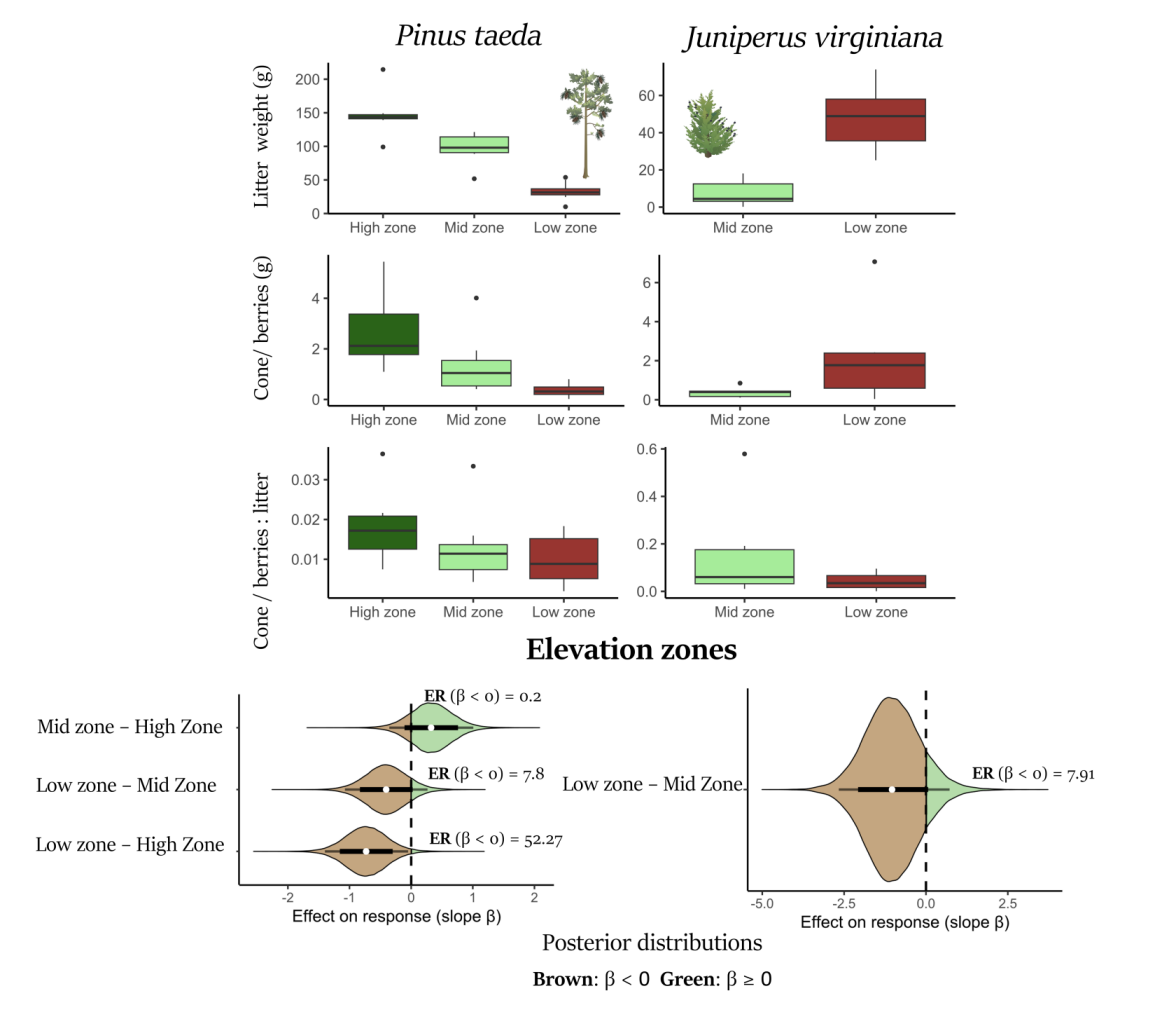
Higher elevation areas have lower salinity values than lower elevation areas. Upper panels: Boxplots show (row 1) total litter weight (g), (row 2) total cone/berry weight (g), and (row 3) the ratio of cone/berry weight to litter weight per forest plot over the two-year sampling period for Pinus taeda (left) and Juniperus virginiana (right) across high, mid, and low elevation zones (J. virginiana present only in mid and low zones). Lower panels: Posterior probability distributions of the effect of forest zone contrasts on the cone/berry:litter ratio (relative investment in reproductive structures relative to litter production) for Pinus taeda (left; three pairwise contrasts among zones) and Juniperus virginiana (right; low vs. mid zone). Brown indicates β < 0 and green indicates β ≥ 0; points represent posterior medians, thick segments the 80% credibility intervals, thin lines the 95% credibility intervals, and evidence ratios (ER) summarize support for β < 0.

## Description

Plants continually re‑balance their resource allocation in response to environmental cues, weighing trade‑offs between growth, reproduction, and survival (Poorter et al., 2012). For example, reproduction incurs costs, illustrated by increased mortality rates after reproductive events and size thresholds necessary for reproduction to occur (Enquist & Niklas, 2002).

Seeds lie at the heart of this investment. These reproductive structures protect the embryo inside and provide it with a package of maternal reserves to be used by the emerging seedling until it achieves independence (Westoby et al., 2002; Moles & Westoby, 2004). Because parental provisioning is critical for establishment, seed size is strongly associated with seedling survival (Saatkamp et al., 2018).

However, stressors such as increasing salinity create a resource shortage. Salinity forces plants to close stomata to conserve water, which restricts carbon intake (McDowell et al., 2022). Simultaneously, the metabolic cost of survival increases as plants must divert energy to exclude or compartmentalize toxic ions (Munns & Tester, 2008). This "double squeeze" drastically shrinks the surplus energy available, with the potential to force shifts in allocation trade-offs.

This dynamic is currently playing out in the Mid-Atlantic United States, where saltwater intrusion is exposing coastal forests to novel, stressful conditions. As seawater infiltrates the root zone, trees respond with lower leaf production and foliage loss symptoms of carbon starvation that signal the emergence of “ghost forests” (Gedan & Kirwan, 2019; McDowell et al., 2022). Under such constraints, plants may be forced to adjust their total reproductive investment, shifting the fraction of biomass allocated to seeds versus maintenance.

To understand these shifts, we use seed rain per unit litter production as a proxy for relative reproductive investment. We examine how salinity stress alters coastal forest trees’ investment among competing life‑history functions, testing if the "double squeeze" of carbon starvation reshapes established cost-benefit relationships.

We hypothesized that plants would exhibit an overall decrease in reproductive allocation in more salt-stressed, low elevation forest, which would manifest as a reduction in seed number, size, or both relative to their overall productivity. Additionally, species may vary in this response based on their tolerance, plasticity, and ability to adapt to ongoing salinization, and therefore, we expected to observe species-specific responses in two common coastal tree species, *Pinus taeda* and *Juniperus virginiana* .

Across the study site, the two tree species exhibited contrasting productivity trends in response to salinity gradients, yet both showcased similar reductions in relative investment on reproductive structures. *Pinus taeda* showed reduced weights of collection for both cones and litter across the salinity gradient (Fig.1 upper panel **)** . The relative allocation to seed structures was significantly lower in low elevation, high-salinity zones compared to high-elevation, low-salinity zones, a difference supported by credibility intervals that did not include zero. While a comparison between mid- and high-salinity areas showed more uncertainty (i.e. credibility intervals including zero), the evidence ratio still strongly suggested decreased reproductive investment at higher salinity levels ( Fig. 1 lower panel).

*Juniperus virginiana* displayed a positive association between salinity and productivity, exhibiting higher weights of both seed and litter fall in areas of greater salinity. However, enhanced productivity at high salinity did not translate into a proportional increase in reproductive investment. Instead, *Juniperus virginiana* showed a decrease in the relative resource allocation to seeds despite increases in overall growth and seed production, suggesting a non-isometric scaling relationship between these processes. While the effect of salinity on the relative reproductive investment of *Juniperus virginiana* was characterized by greater uncertainty, as indicated by credibility intervals that included zero ( Fig. 1, right panel), the evidence ratio still favored a decrease in relative investment by a factor of 7.91. This suggests that despite the uncertainty in the credibility interval, a reduction in reproductive investment with increasing salinity is very likely for *Juniperus virginiana* .

Our research reveals significant variation in productivity and reproductive allocation across a coastal salinity gradient, highlighting contrasting patterns between two tree species. In higher salinity conditions, *Pinus taeda* exhibited decreased reproductive allocation, suggesting a potential shift towards greater investment in growth and survival under stressful conditions. Changes in cone weight could reflect shifts in seed number or size, or a reduced investment in the energetically costly cone structure. As soil salinity increases, *Pinus taeda* trees adjust their allocation schedules to accommodate novel environmental conditions, prioritizing allocation to growth and maintenance.

Conversely, seed weight and litter production in *Juniperus virginiana* showed an increase when in more saline conditions *. Juniperus virginiana,* a shade-intolerant species, benefits from increased light availability in canopy gaps, leading to enhanced photosynthetic activity (Ornsbee et al., 1976). Productivity increased in highly salinized areas, in spite of the greater salinity, hinting at the importance of light availability for this understory tree. Yet, even so, an increase in productivity did not deliver an increase in relative investment to reproductive structures, but the opposite; there was a decrease in relative investment toward reproductive structures in more salt-stressed forest in *Juniperus virginiana* as well. This apparent discrepancy suggests a shift in investment to favor future, instead of current reproductive output, a canonical tradeoff in life history theory.

This observed pattern is crucial, as it sheds light on the impact of saltwater intrusion on a fundamental aspect of forest dynamics, regeneration. Are the changes in seed collection a result of changes in parental provisioning of resources? If so, how might this affect seedling performance? The findings presented here provide valuable insights into another dimension of the impact of salinization on coastal ecosystems.

By highlighting the response of trees to ecological gradients created by sea level rise, we document the variety of ways species are responding to climate change and reveal how species’ responses in reproductive allocation scale to community dynamics such as productivity and regeneration. Studying plant responses and modes of adaptation will enhance our ability to predict patterns of forest change and develop new forest management approaches for the future.

## Methods

Seed rain was measured at a forested site within Virginia's Nature Conservancy Brownsville Preserve, part of the Virginia Coast Reserve Long-Term Ecological Research site. Three fixed-station baskets (0.25 m x 0.25 m) were deployed within twenty-four 20 x 20 m plots across the salinity gradient that spans less than one kilometer. Litter and seeds were collected monthly for 18 months, from May 2021 through December 2022. Each collection was sorted to species and structure (litter or seeds/cones) based on morphological identification. Leaf litter of each species served as a proxy for production, while seeds in litter represented seed rain. For *Pinus taeda* , pine cone weight served as a proxy for reproductive allocation. Three salinity zones were defined based on tree canopy health and understory vegetation composition, which were later confirmed by repeated soil salinity measurements. Areas closer to the marsh exhibited higher salinization, more frequent flooding, more open canopies, and a denser presence of sub-canopy shrubs such as *Morella cerifera* or the grasses *Phragmites australis, Spartina patens,* and *Distichlis spicata* . These ecological differences defined three zones: high, mid, and low elevation (Sward et al. 2023; Langston et al., 2024). Eight plots were located in each of these three zones. The elevation gradient correlates with salinity, with low lying areas showing high salinity values.

To explore covariation patterns between relative investment in seeds across the salinity gradient, Bayesian linear regression models were implemented for each tree species. These models evaluated the shift in seed weight per unit of litter production across the gradient, addressing changes in allocational schemes. This variable followed a lognormal distribution due to its natural lower bound of zero. Normal priors, centered at zero with two standard deviations as dispersion measures, were defined as weakly informative priors. A posterior distribution is the probability distribution of a model parameter after updating the prior information with the observed data; it summarizes the range of parameter values compatible with both and assigns a probability to each value, allowing us to directly evaluate the probability of specific parameter values (e.g., β<0). On this basis, we quantified evidence ratios to measure the strength of statistical support, interpreted as how many times more likely a hypothesis is compared to its alternative.

To address the potential influence of stand density and tree size on overall reproductive allocation, we tested competing models with and without the basal area of the focal species at the plot scale. The best-fitting model, selected based on leave-one-out validation metrics (LOO), included only the zone as a predictor. These patterns were consistent throughout the two-year sampling period.

## References

[R1] Enquist Brian J., Niklas Karl J. (2002). Global Allocation Rules for Patterns of Biomass Partitioning in Seed Plants. Science.

[R2] Kirwan Matthew L., Gedan Keryn B. (2019). Sea-level driven land conversion and the formation of ghost forests. Nature Climate Change.

[R3] Langston Amy K., Smith Alexander J., Gedan Keryn B., Kirwan Matthew L. (2025). Ecosystem structure and salinity thresholds in retreating coastal forests along the Mid-Atlantic, USA. Climatic Change.

[R4] McDowell Nate G., Ball Marilyn, Bond‐Lamberty Ben, Kirwan Matthew L., Krauss Ken W., Megonigal J. Patrick, Mencuccini Maurizio, Ward Nicholas D., Weintraub Michael N., Bailey Vanessa (2022). Processes and mechanisms of coastal woody‐plant mortality. Global Change Biology.

[R5] Metz Johannes, Liancourt Pierre, Kigel Jaime, Harel Danny, Sternberg Marcelo, Tielbörger Katja (2010). Plant survival in relation to seed size along environmental gradients: a long‐term study from semi‐arid and Mediterranean annual plant communities. Journal of Ecology.

[R6] MOLES ANGELA T., WESTOBY MARK (2004). Seedling survival and seed size: a synthesis of the literature. Journal of Ecology.

[R7] Munns Rana, Tester Mark (2008). Mechanisms of Salinity Tolerance. Annual Review of Plant Biology.

[R8] Ormsbee, P., Bazzaz, F. A., & Boggess, W. R. (1976). Physiological ecology of *Juniperus virginiana* in oldfields. *Oecologia* , *23* (2), 75–82. 10.1007/BF00351216 28309214

[R9] Poorter Hendrik, Niklas Karl J., Reich Peter B., Oleksyn Jacek, Poot Pieter, Mommer Liesje (2011). Biomass allocation to leaves, stems and roots: meta‐analyses of interspecific variation and environmental control. New Phytologist.

[R10] Saatkamp Arne, Cochrane Anne, Commander Lucy, Guja Lydia K. , Jimenez‐Alfaro Borja, Larson Julie, Nicotra Adrienne , Poschlod Peter, Silveira Fernando A. O., Cross Adam T., Dalziell Emma L., Dickie John, Erickson Todd E., Fidelis Alessandra, Fuchs Anne, Golos Peter J., Hope Michael, Lewandrowski Wolfgang, Merritt David J., Miller Ben P., Miller Russell G. , Offord Catherine A., Ooi Mark K. J., Satyanti Annisa , Sommerville Karen D., Tangney Ryan, Tomlinson Sean , Turner Shane, Walck Jeffrey L. (2018). A research agenda for seed‐trait functional ecology. New Phytologist.

[R11] Sward Rheya, Philbrick Abigail, Morreale Jonah, Baird Cora Johnston, Gedan Keryn (2023). Shrub expansion in maritime forest responding to sea level rise. Frontiers in Forests and Global Change.

[R12] Westoby Mark, Falster Daniel S., Moles Angela T., Vesk Peter A., Wright Ian J. (2002). Plant Ecological Strategies: Some Leading Dimensions of Variation Between Species. Annual Review of Ecology and Systematics.

[R13] Yan Shipeng, Chong Peifang, Zhao Ming (2022). Effect of salt stress on the photosynthetic characteristics and endogenous hormones, and: A comprehensive evaluation of salt tolerance in
*Reaumuria soongorica*
seedlings. Plant Signaling & Behavior.

